# Supplementary material for: IL-36 signaling as a drug target in Crohn’s disease patients with IL36RN mutations
Source: EMBO Mol Med. 2025 May 30;17(7):1539–55. doi: 10.1038/s44321-025-00245-z (PMC12254353; doi:10.1038/s44321-025-00245-z)
Supplement: Supplementary file 1 — Appendix [file 44321_2025_245_MOESM1_ESM.pdf]

APPENDIX Hecker et al.

Table of Content

Appendix Figure S1: Sanger Sequencing of sorted cells of the IL-36RA patient **- page 2**

Appendix Figure S2: Deep immune cell profiling of the IL-36RA patient by mass cytometry **– page 2**

Appendix Figure S3: Analysis of the T cell compartment of the IL-36RA patient by mass cytometry **– page 3**

Appendix Figure S4: Cytokine profile in the serum of the IL-36RA patient **– page 3**

Appendix Figure S5: Myeloid cells are the main responders to IL-36α stimulation in the blood **– page 4**

Appendix Figure S6: MRI scans of the IL-36RA patient before and during spesolimab therapy **– page 5**

Appendix Figure S7: Changes in the immune cell composition of the IL-36RA patient during spesolimab therapy **– page 6**

Appendix Figure S8: Changes in serum cytokine levels of the IL-36RA patient during spesolimab therapy **– page 7**

Appendix Figure S9: RNA sequencing of intestinal biopsies of the IL-36RA patient **– page 8**

Appendix Figure S10: Gating strategy for the analysis of different immune cell subsets in LPMCs by flow cytometry **– page 9**

Appendix Table S1: Treatment history of the IL-36RA patient **– page 10**

Appendix Table S2: SES-CD score and weight of the IL-36RA patient during spesolimab therapy **– page 10**

Appendix Table S3: p-values from the statistical analysis conducted in this study **– page 11**

Appendix Table S4: Sample IDs of control samples for bulk RNA sequencing – **page 12**

Appendix Table S5: Authorlist TRR241 IBDome Consortium **– page 12**

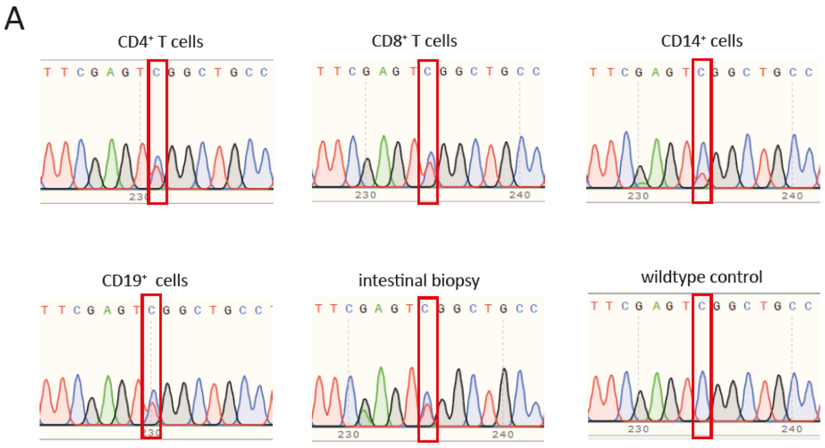

Appendix Figure S1: Sanger Sequencing of sorted cells of the IL-36RA patient

(A) Sanger sequencing of sorted CD4+, CD8+, CD14+ and CD19+ cells and of an intestinal biopsy of the *IL36RN*-mutated patient as well as of PBMCs of an unrelated control (wildtype control). The red square indicates the position of the mutation (c.338C) in the *IL36RN* gene.

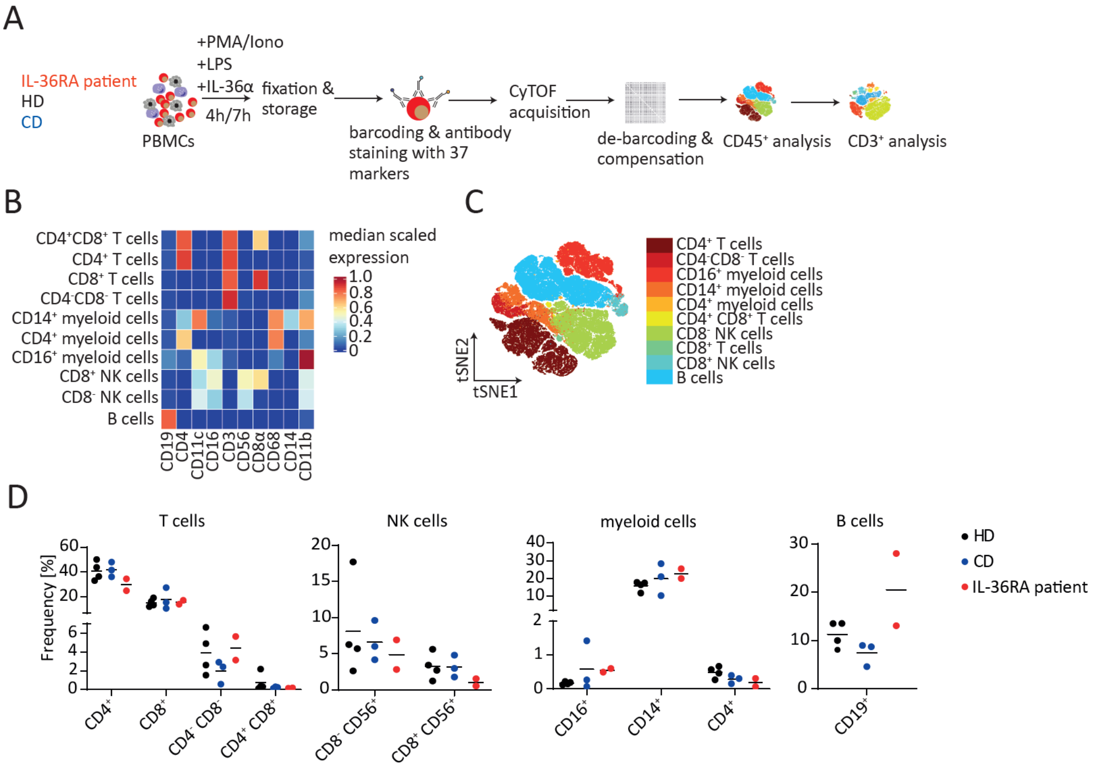

Appendix Figure S2: Deep immune cell profiling of the IL-36RA patient by mass cytometry

(A) Schematic summary of the immune cell characterization of the *IL36RN*-mutated patient (IL-36RA patient) by mass cytometry. PBMCs of the IL-36RA patient at two different time points, Crohn's disease patients (CD) and healthy donors (HD) were *in vitro* stimulated with phorbol 12-myristate 13-acetate (PMA)/ionomycin (Iono) or lipopolysaccharide (LPS) for 4 h or with IL-36α for 7 h and fixed. Subsequently, PBMCs were stained with 37 metal-conjugated antibodies and acquired by mass cytometry (CyTOF). Resulting FCS files were de-barcoded, compensated and gated on CD45+ cells. Following, reduced-dimensional (2D) t-SNE maps were generated, clusters were identified and differential expression and abundance was analyzed. **(B)** Heat map showing the expression of 10 selected markers used for clustering of CD45+ PBMCs. **(C)** t-SNE plot colored by the 10 identified clusters. **(D)** Frequency of the 10 different clusters in unstimulated PBMCs. Line in the plots indicates the median. HD: n = 4, CD: n= 3, IL-36RA patient: n= 2 (same patient, different time points).

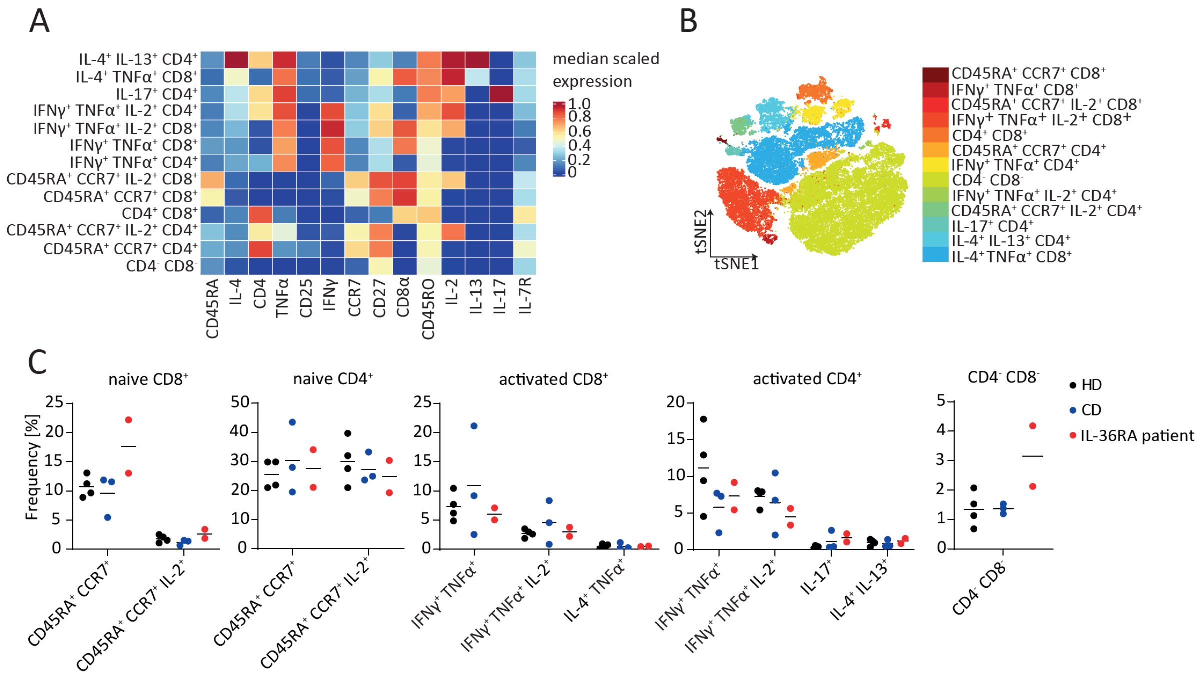

Appendix Figure S3: Analysis of the T cell compartment of the IL-36RA patient by mass cytometry

(A) Heat map showing the expression of 14 selected markers used for clustering of CD3⁺ peripheral blood mononuclear cells (PBMCs). **(B)** t-SNE plot colored by the 13 identified clusters. **(C)** Frequency of the 13 different clusters in phorbol 12-myristate 13-acetate (PMA)/ionomycin (Iono)-stimulated PBMCs of the *IL36RN*-mutated patient (IL-36RA patient) at two different time points, Crohn's disease patients (CD) and healthy donors (HD). Line in the plots indicates the median. HD: n = 4, CD: n= 3, IL-36RA patient: n= 2 (same patient, different time points).

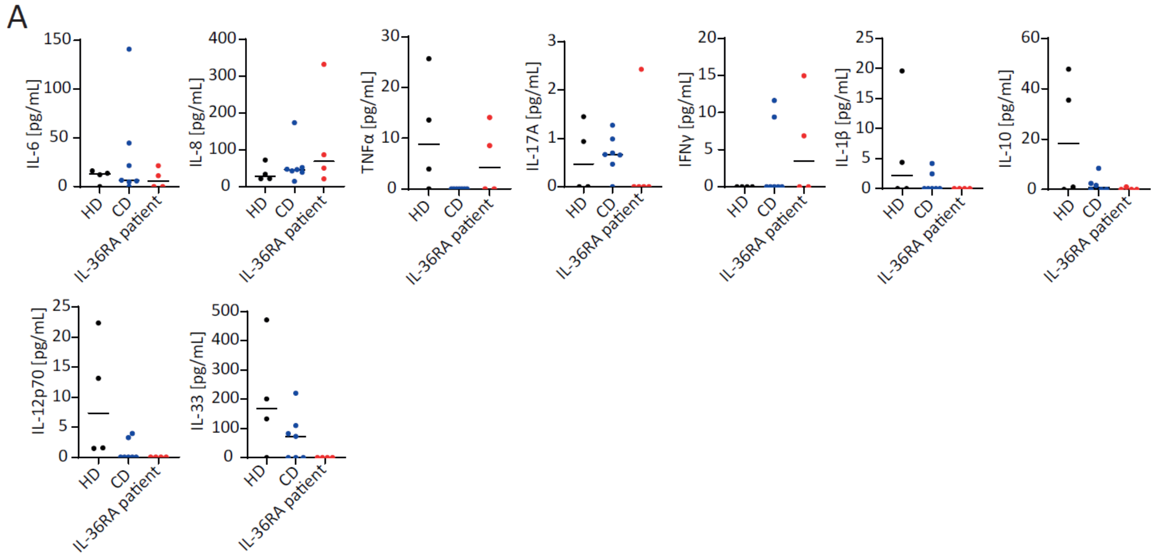

Appendix Figure S4: Cytokine profile in the serum of the IL-36RA patient

(A) The concentration of pro-inflammatory cytokines in the serum of healthy donors (HD), Crohn's disease patients (CD) and the *IL36RN*-mutated patient (IL-36RA patient) at different time points. HD: n = 4, CD: n= 7, IL-36RA patient: n= 4 (same patient, different time points).

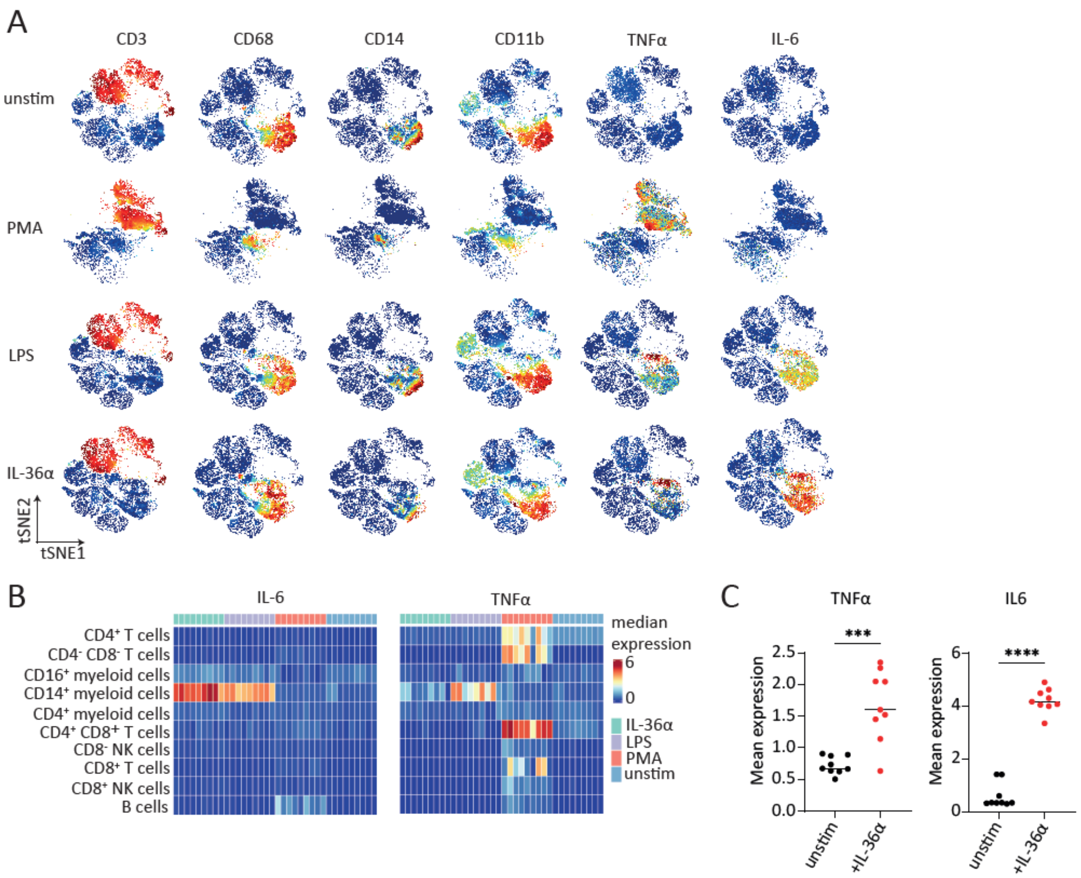

Appendix Figure S5: Myeloid cells are the main responders to IL-36α stimulation in the blood

(A-C) Peripheral blood mononuclear cells (PBMCs) of Crohn's disease patients and healthy donors (n= 9) were stimulated *in vitro* with phorbol 12-myristate 13-acetate (PMA)/ionomycin (Iono) or lipopolysaccharide (LPS) for 4 h or with IL-36α for 7 h. Subsequently, the samples were analyzed by mass cytometry. **(A)** t-SNE plots colored by the expression of selected markers. **(B)** Heatmaps showing the expression of IL-6 and TNFα in different subsets identified in PBMCs. **(C)** Mean expression of TNFα and IL-6 in $CD14^+$ myeloid cells. Statistical significance was determined by paired t test. ****$p<0.0001$, ***$p<0.001$, **$p<0.01$, *$p<0.05$.

A

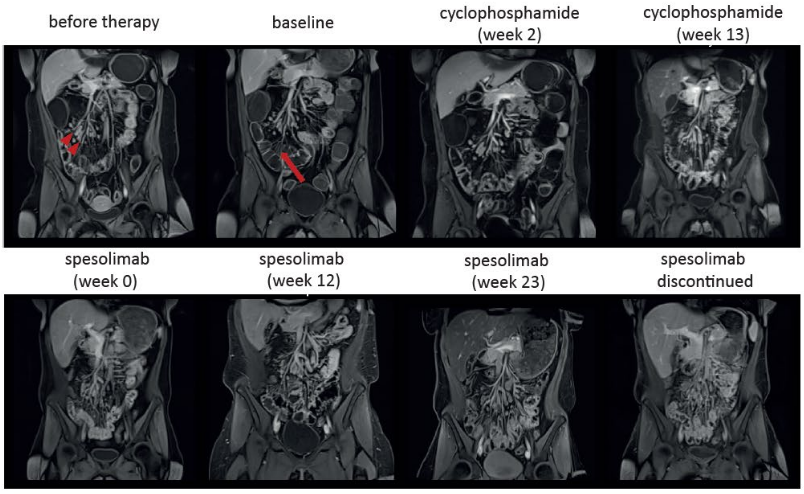

Appendix Figure S6: MRI scans of the IL-36RA patient before and during spesolimab therapy
(A) T1 weighted, fat-saturated MRI after i.v. contrast administration in coronal plane. There is a moderately pronounced mesenteric lymphadenopathy (arrowheads) and a Comb-sign in the mid-abdomen (arrows) with otherwise unremarkable visualization of the mesenteric vascular tree over the course of 12 months. Segmentally inflamed bowel loops were not seen at any time.

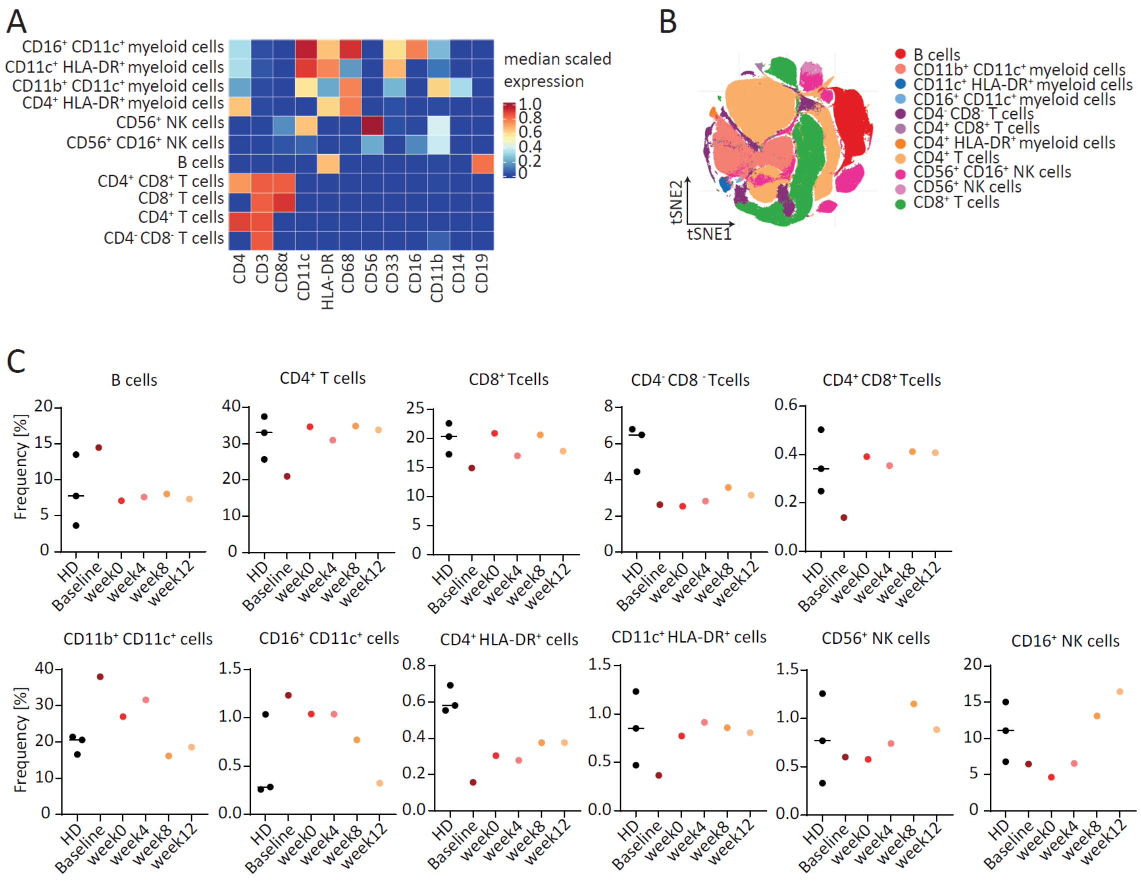

Appendix Figure S7: Changes in the immune cell composition of the IL-36RA patient during spesolimab therapy
(A-C) Peripheral blood mononuclear cells (PBMCs) of the *IL36RN*-mutated patient (IL-36RA patient) at different time points before and during spesolimab therapy and of healthy donors (HD) were *in vitro* stimulated with phorbol 12-myristate 13-acetate (PMA)/ionomycin (Iono) or lipopolysaccharide (LPS) for 4 h or with IL-36α for 7 h and subsequently analyzed by mass cytometry **(A)** Heat map showing the expression of 12 selected markers used for clustering of $CD45^+$ PBMCs. **(B)** t-SNE plot colored by the 11 identified clusters. **(C)** Frequency of the 13 different clusters in unstimulated PBMCs. Line in the plots indicates the median. HD: n = 3, IL-36RA patient: n = 1.

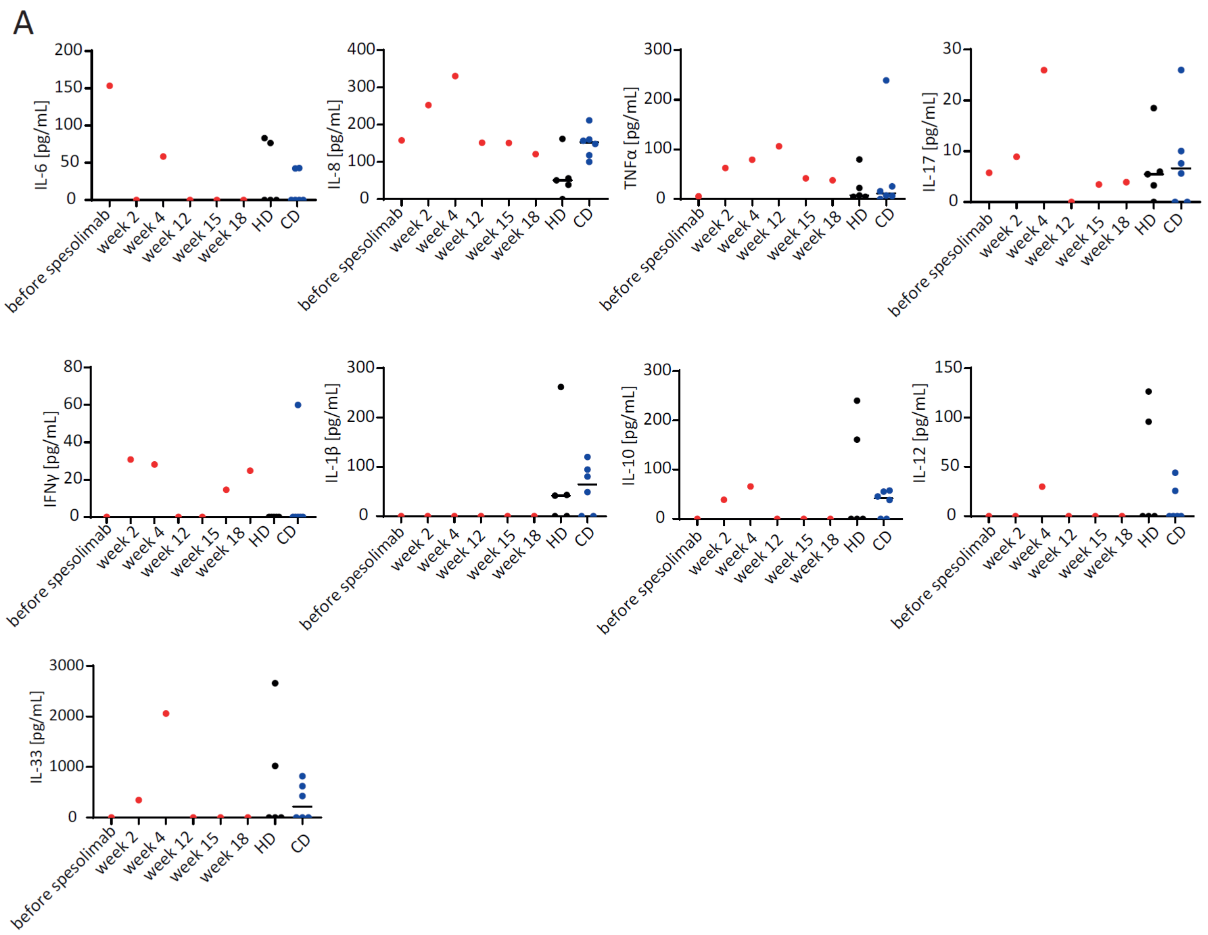

Appendix Figure S8: Changes in serum cytokine levels of the IL-36RA patient during spesolimab therapy

(A) The concentration of pro-inflammatory cytokines in the serum of healthy donors (HD), Crohn's disease patients (CD) and the *IL36RN*-mutated patient (IL-36RA patient) at different time points before and during spesolimab therapy. HD: n = 5, CD: n= 6, IL-36RA patient: n= 1.

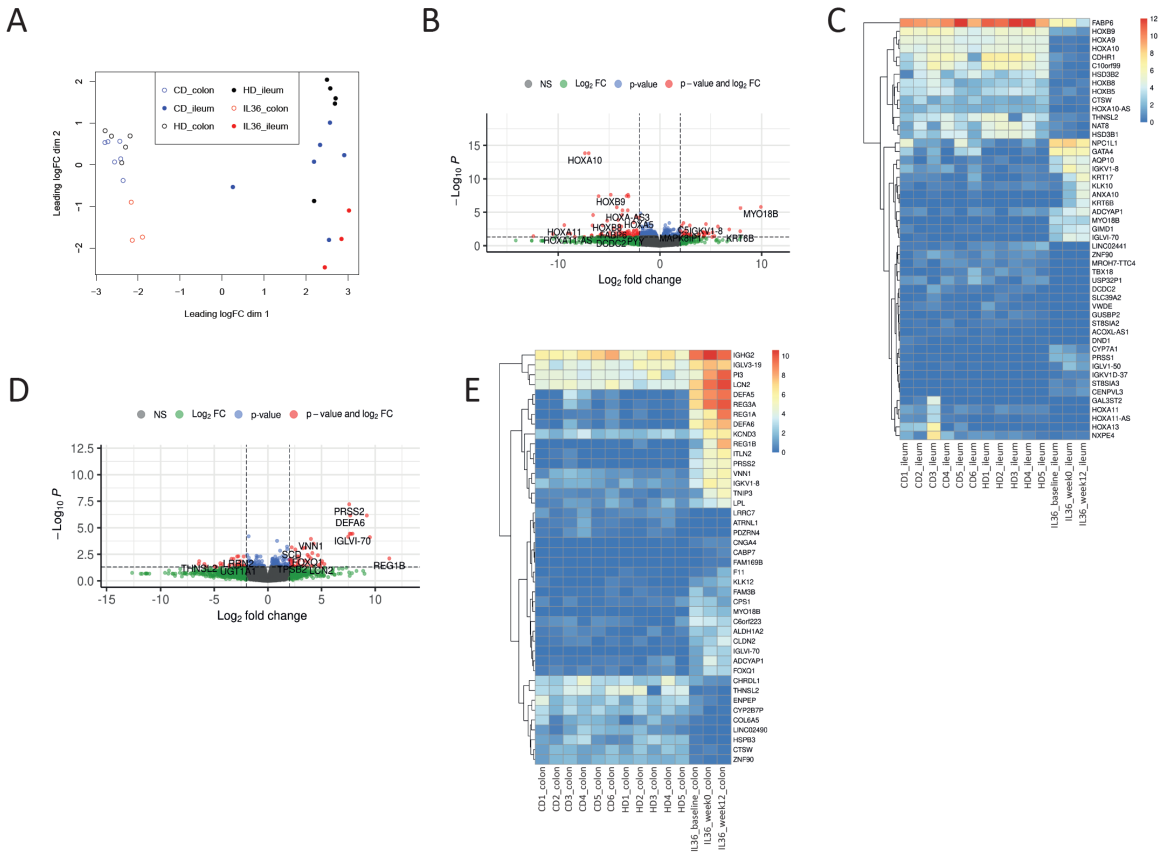

Appendix Figure S9: RNA sequencing of intestinal biopsies of the IL-36RA patient

(A) MDS plot of RNA sequencing data from intestinal biopsies of the *IL36RN*-mutated patient (IL-36RA patient), Crohn's disease patients (CD) and healthy donors (HD). **(B)** Volcano plot of RNA sequencing analysis of ileal biopsies of the IL-36RA patient compared to CD patients (padjusted<0.05, log_2FC cutoff = 2) **(C)** Heatmap showing the normalized expression of selected genes in ileal biopsies (padjusted<0.05, log_2FC cutoff = 4). **(D)** Volcano plot of RNA sequencing analysis of colonic biopsies of the IL-36RA patient compared to CD patients (padjusted<0.05, log_2FC cutoff = 2). **(E)** Heatmap showing the normalized expression of selected genes in colonic biopsies (padjusted<0.05, log_2FC cutoff = 3). HD: n = 5, CD: n= 6, IL-36RA patient: n= 3 (same patient, different time points).

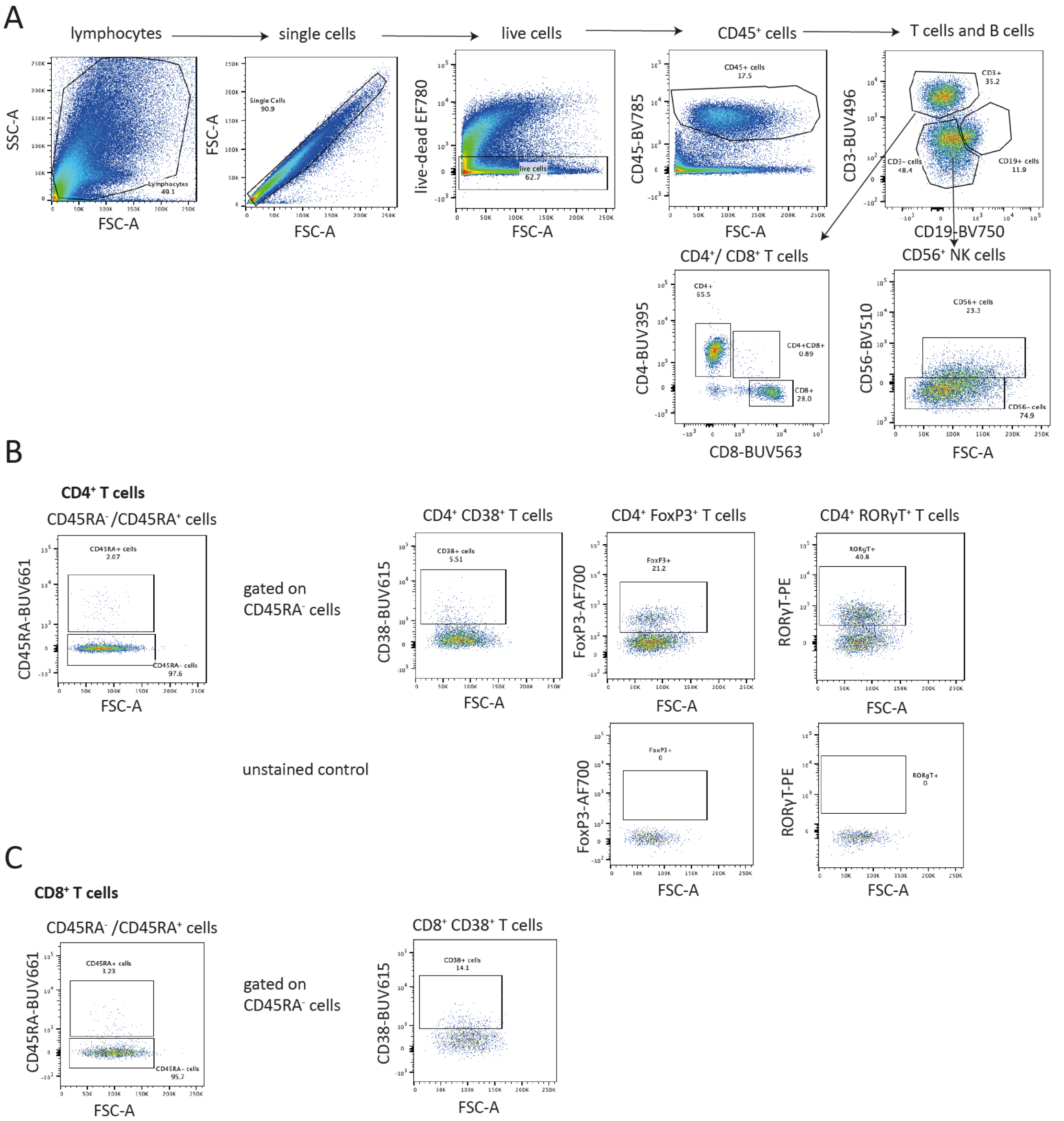

Appendix Figure S10: Gating strategy for the analysis of different immune cell subsets in LPMCs by flow cytometry

(A) Gating strategy for the identification of CD4⁺ T cells, CD8⁺ T cells, CD19⁺ B cells and CD56⁺ NK cells in lamina propria mononuclear cells (LPMCs) isolated from biopsies of the ileum and colon of the *IL36RN*-mutated patient (IL-36RA patient) and other Crohn's disease (CD) patients. **(B)** Identification of different subsets among CD4⁺ T cells in LPMCs of the IL-36RA patient and other CD patients. **(C)** Identification of different subsets among CD8⁺ T cells in LPMCs of the IL-36RA patient and other CD patients.

Appendix Table S1: Treatment history of the IL-36RA patient

Age [years]	Treatment	Reason for discontinuation
19	Azathioprine	Intolerance: vomitting
20	6-mercaptopurine	Intolerance: pancreatitis
20	Adalimumab	Secondary treatment failure
20	Infliximab	Secondary treatment failure
20	Andante (anti-IL-6)	Treatment failure
20	Methotrexate	Primary treatment failure
20	Protective ileostomy	
21	Ustekinumab	Secondary treatment failure
21	Vedolizumab	Secondary treatment failure
21-22	Certolizumab	Secondary treatment failure
22-23	Methotrexate	Treatment failure
22	Ileal segment resection	
23-24	Golimumab	Treatment failure
25	Autologous stem cell transplantation	
25	Ileocecal resection	
25-27	Adalimumab	Tertiary loss of response
27	Infliximab	Fulminant allergic reaction
27	Golimumab	Therapy failure
27-28	Upadacitinib	Secondary treatment failure
28	Methotrexate	
28	Protective ileostomy	
29	Spesolimab therapy	Discontinuation of therapy due to small perianal abscess
29	Risankizumab	

Appendix Table S2: SES-CD score and weight of the IL-36RA patient during spesolimab therapy

Table showing the weight and the Simple Endoscopic Score for Crohn's Disease (SES-CD) of the *IL36RN*-mutated patient before and during treatment with spesolimab.

Timepoint	Weight [kg]	SES-CD
Baseline	Not assessed	12
Week 0 (after cyclophosphamide)	56,4	9
Week 2 of spesolimab treatment	56,4	Not assessed
Week 4 of spesolimab treatment	54,7	Not assessed
Week 8 of spesolimab treatment	56,0	Not assessed
Week 12 of spesolimab treatment	55,0	6
Week 15 of spesolimab treatment	54,0	Not assessed
Week 18 of spesolimab treatment	53,9	Not assessed

Appendix Table S3: p-values from the statistical analysis conducted in this study

Figure panel and comparison	Statistical test and p-value
Figure 1C	Ordinary One-way ANOVA, Tukey's multiple comparisons test
WT vs. S113L	0.0002
WT vs. untrans.	<0.0001
WT vs. H_2O	<0.0001
S113L vs. untrans.	0.0161
S113L vs. H_2O	0.0125
untrans vs. H_2O	0.9995
Figure 1D	Ordinary One-way ANOVA. Tukey's multiple comparisons test
WT vs. S113L	<0.0001
WT vs. untrans.	<0.0001
S113L vs. untrans.	0.7577
Figure 3B	Ordinary One-way ANOVA. Tukey's multiple comparisons test
WT vs. S113L	0.0011
WT vs. P76L	0.008
WT vs. L133I	0.5912
WT vs. H_2O	<0.0001
WT vs. untrans.	<0.0001
S113L vs. P76L	0.9715
S113L vs. L133I	0.0637
S113L vs. H_2O	0.0648
S113L vs. untrans.	0.052
P76L vs. L133I	0.2811
P76L vs. H_2O	0.0105
P76L vs. untrans.	0.0081
L133I vs. H_2O	<0.0001
L133I vs. untrans.	<0.0001
H_2O vs. untrans.	>0.9999
Figure 3C	Ordinary One-way ANOVA. Tukey's multiple comparisons test
WT vs. S113L	<0.0001
WT vs. P76L	<0.0001
WT vs. L133I	<0.0001
WT vs. untrans.	<0.0001
S113L vs. P76L	>0.9999
S113L vs. L133I	<0.0001
S113L vs. untrans.	0.9994
P76L vs. L133I	<0.0001
P76L vs. untrans.	0.9993
L133I vs. untrans.	<0.0001
Figure EV1C	Ordinary One-way ANOVA. Tukey's multiple comparisons test
TNFα unstim. vs. spesolimab	>0.9999
TNFα unstim. vs. IL-36α	<0.0001

TNFα unstim. vs. IL-36α + spesolimab	<0.0001
TNFα spesolimab vs. IL-36α	<0.0001
TNFα spesolimab vs. IL-36α + spesolimab	<0.0001
TNFα IL-36α vs. IL-36α + spesolimab	<0.0001
IL-6 unstim. vs. spesolimab	>0.9999
IL-6 unstim. vs. IL-36α	<0.0001
IL-6 unstim. vs. IL-36α + spesolimab	<0.0001
IL-6 spesolimab vs. IL-36α	<0.0001
IL-6 spesolimab vs. IL-36α + spesolimab	<0.0001
IL-6 IL-36α vs. IL-36α + spesolimab	<0.0001
IL-23 unstim. vs. spesolimab	>0.9999
IL-23 unstim. vs. IL-36α	<0.0001
IL-23 unstim. vs. IL-36α + spesolimab	0.0300
IL-23 spesolimab vs. IL-36α	<0.0001
IL-23 spesolimab vs. IL-36α + spesolimab	0.0300
IL-23 IL-36α vs. IL-36α + spesolimab	<0.0001

Appendix Table S4: Sample IDs of control samples for bulk RNA sequencing

**Sample ID used in this paper**	**Sample ID in IBDome database**
HD1_ileum	ibdome_berlin:20008a500
HD1_colon	ibdome_berlin:20008a501
HD2_ileum	ibdome_berlin:20008a502
HD2_colon	ibdome_berlin:20008a503
HD3_ileum	ibdome_berlin:20008a505
HD3_colon	ibdome_berlin:20008a506
HD4_ileum	ibdome_berlin:20008a507
HD4_colon	ibdome_berlin:20008a508
HD5_ileum	ibdome_berlin:20008a511
HD5_colon	ibdome_berlin:20008a512
CD1_ileum	ibdome_berlin:20008a409
CD1_colon	ibdome_berlin:20008a410
CD2_ileum	ibdome_berlin:20008a416
CD2_colon	ibdome_berlin:20008a417
CD3_ileum	ibdome_berlin:20008a420
CD3_colon	ibdome_berlin:20008a421
CD4_ileum	ibdome_berlin:20008a430
CD4_colon	ibdome_berlin:20008a431
CD5_ileum	ibdome_berlin:20008a453
CD5_colon	ibdome_berlin:20008a454
CD6_ileum	ibdome_berlin:20008a457
CD6_colon	ibdome_berlin:20008a458

Appendix Table S5: Authorlist TRR241 IBDome Consortium

**Author**	**Affiliation**
Imke Atreya	Department of Medicine 1, Friedrich-Alexander University, Erlangen, Germany

Raja Atreya	Department of Medicine 1, Friedrich-Alexander University, Erlangen, Germany
Petra Bacher	Institute of Clinical Molecular Biology, Christian-Albrecht University of Kiel, Kiel, Germany;<br>Institute of Immunology, Christian-Albrecht University of Kiel and UKSH Schleswig-Holstein, Kiel, Germany
Christoph Becker	Department of Medicine 1, Friedrich-Alexander University, Erlangen, Germany
Christian Bojarski	Charité – Universitätsmedizin Berlin, corporate member of Freie Universität Berlin and Humboldt-Universität zu Berlin, Department of Gastroenterology, Infectious Diseases and Rheumatology, Berlin, Germany
Nathalie Britzen-Laurent	Department of Medicine 1, Friedrich-Alexander University, Erlangen, Germany
Caroline Bosch-Voskens	Department of Medicine 1, Friedrich-Alexander University, Erlangen, Germany
Hyun-Dong Chang	Deutsches Rheuma-Forschungszentrum, ein Institut der Leibniz-Gemeinschaft, Berlin, Germany
Andreas Diefenbach	Charité – Universitätsmedizin Berlin, corporate member of Freie Universität Berlin and Humboldt-Universität zu Berlin, Institute of Microbiology, Infectious Diseases and Immunology, Berlin, Germany
Claudia Günther	Department of Medicine 1, Friedrich-Alexander University, Erlangen, Germany
Ahmed N. Hegazy	Charité – Universitätsmedizin Berlin, corporate member of Freie Universität Berlin and Humboldt-Universität zu Berlin, Department of Gastroenterology, Infectious Diseases and Rheumatology, Berlin, Germany
Kai Hildner	Department of Medicine 1, Friedrich-Alexander University, Erlangen, Germany
Christoph S.N. Klose	Charité – Universitätsmedizin Berlin, corporate member of Freie Universität Berlin and Humboldt-Universität zu Berlin, Institute of Microbiology, Infectious Diseases and Immunology, Berlin, Germany

Kristina Koop	Department of Medicine 1, Friedrich-Alexander University, Erlangen, Germany
Susanne Krug	Charité – Universitätsmedizin Berlin, corporate member of Freie Universität Berlin and Humboldt-Universität zu Berlin, Department of Gastroenterology, Infectious Diseases and Rheumatology, Berlin, Germany
Anja A. Kühl	Charité – Universitätsmedizin Berlin, corporate member of Freie Universität Berlin and Humboldt-Universität zu Berlin, iPATH.Berlin, Berlin, Germany
Moritz Leppkes	Department of Medicine 1, Friedrich-Alexander University, Erlangen, Germany
Rocío López-Posadas	Department of Medicine 1, Friedrich-Alexander University, Erlangen, Germany
Leif S.-H. Ludwig	Berlin Institute of Health at Charité – Universitätsmedizin Berlin, Berlin, Germany;<br>Max Delbrück Center for Molecular Medicine in the Helmholtz Association [MDC], Berlin Institute for Medical Systems Biology [BIMSB], Berlin, Germany
Clemens Neufert	Department of Medicine 1, Friedrich-Alexander University, Erlangen, Germany
Markus Neurath	Department of Medicine 1, Friedrich-Alexander University, Erlangen, Germany
Jay Patankar	Department of Medicine 1, Friedrich-Alexander University, Erlangen, Germany
Magdalena Prüß	Charité – Universitätsmedizin Berlin, corporate member of Freie Universität Berlin and Humboldt-Universität zu Berlin, Department of Gastroenterology, Infectious Diseases and Rheumatology, Berlin, Germany
Andreas Radbruch	Deutsches Rheuma-Forschungszentrum, ein Institut der Leibniz-Gemeinschaft, Berlin, Germany
Chiara Romagnani	Charité – Universitätsmedizin Berlin, corporate member of Freie Universität Berlin and Humboldt-Universität zu Berlin, Department of Gastroenterology, Infectious Diseases and Rheumatology, Berlin, Germany;

	Deutsches Rheuma-Forschungszentrum, ein Institut der Leibniz-Gemeinschaft, Berlin, Germany
Francesca Ronchi	Charité – Universitätsmedizin Berlin, corporate member of Freie Universität Berlin and Humboldt-Universität zu Berlin, Institute of Microbiology, Infectious Diseases and Immunology, Berlin, Germany
Ashley Sanders	Charité – Universitätsmedizin Berlin, corporate member of Freie Universität Berlin and Humboldt-Universität zu Berlin, Department of Gastroenterology, Infectious Diseases and Rheumatology, Berlin, Germany;<br>Max Delbrück Center for Molecular Medicine in the Helmholtz Association [MDC], Berlin Institute for Medical Systems Biology [BIMSB], Berlin, Germany
Alexander Scheffold	Institute of Clinical Molecular Biology, Christian-Albrecht University of Kiel, Kiel, Germany
Jörg-Dieter Schulzke	Charité – Universitätsmedizin Berlin, corporate member of Freie Universität Berlin and Humboldt-Universität zu Berlin, Department of Gastroenterology, Infectious Diseases and Rheumatology, Berlin, Germany
Michael Schumann	Charité – Universitätsmedizin Berlin, corporate member of Freie Universität Berlin and Humboldt-Universität zu Berlin, Department of Gastroenterology, Infectious Diseases and Rheumatology, Berlin, Germany
Sebastian Schürmann	Department of Medicine 1, Friedrich-Alexander University, Erlangen, Germany
Britta Siegmund	Charité – Universitätsmedizin Berlin, corporate member of Freie Universität Berlin and Humboldt-Universität zu Berlin, Department of Gastroenterology, Infectious Diseases and Rheumatology, Berlin, Germany
Michael Stürzl	Department of Medicine 1, Friedrich-Alexander University, Erlangen, Germany
Zlatko Trajanoski	Biocenter, Institute of Bioinformatics, Medical University of Innsbruck, Innsbruck, Austria.

Antigoni Triantafyllopoulou	Biocenter, Institute of Bioinformatics, Medical University of Innsbruck, Innsbruck, Austria.;<br>Charité – Universitätsmedizin Berlin, corporate member of Freie Universität Berlin and Humboldt-Universität zu Berlin, Department of Rheumatology and Clinical Immunology, Berlin, Germany
Maximilian Waldner	Department of Medicine 1, Friedrich-Alexander University, Erlangen, Germany
Carl Weidinger	Charité – Universitätsmedizin Berlin, corporate member of Freie Universität Berlin and Humboldt-Universität zu Berlin, Department of Gastroenterology, Infectious Diseases and Rheumatology, Berlin, Germany
Stefan Wirtz	Department of Medicine 1, Friedrich-Alexander University, Erlangen, Germany
Sebastian Zundler	Department of Medicine 1, Friedrich-Alexander University, Erlangen, Germany
