# Supplementary figures and images for: IL-36 signaling as a drug target in Crohn’s disease patients with IL36RN mutations
Source: EMBO Mol Med. 2025 May 30;17(7):1539–55. doi: 10.1038/s44321-025-00245-z (PMC12254353; doi:10.1038/s44321-025-00245-z)

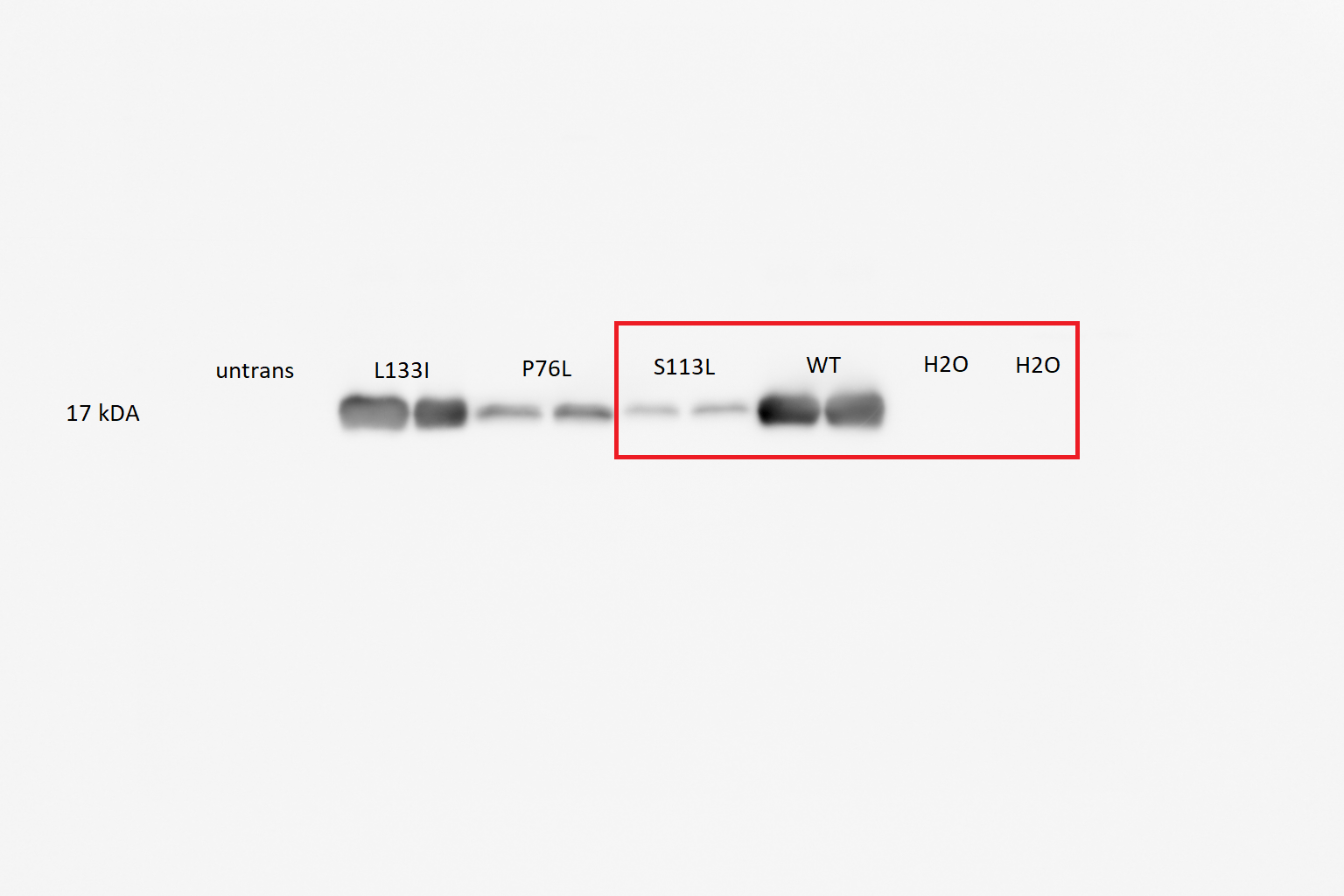

Supplement: Supplementary file 3 — Source data Fig. 1 [file 44321_2025_245_MOESM3_ESM.zip › Figure 1/Figure 1C_IL36RA_mem2.3.tif]

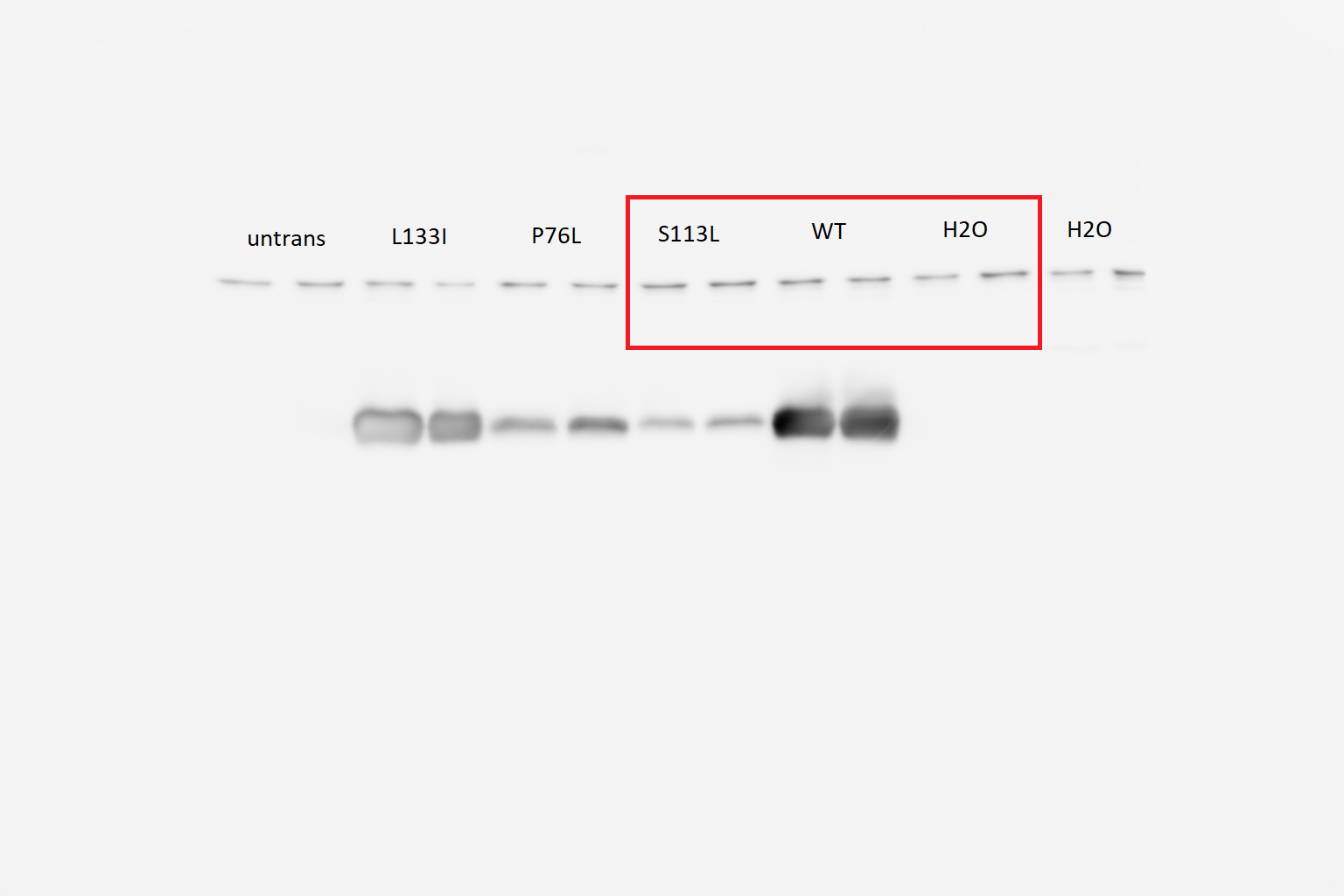

Supplement: Supplementary file 3 — Source data Fig. 1 [file 44321_2025_245_MOESM3_ESM.zip › Figure 1/Figure1C_beta_actin_mem2.3.tif]

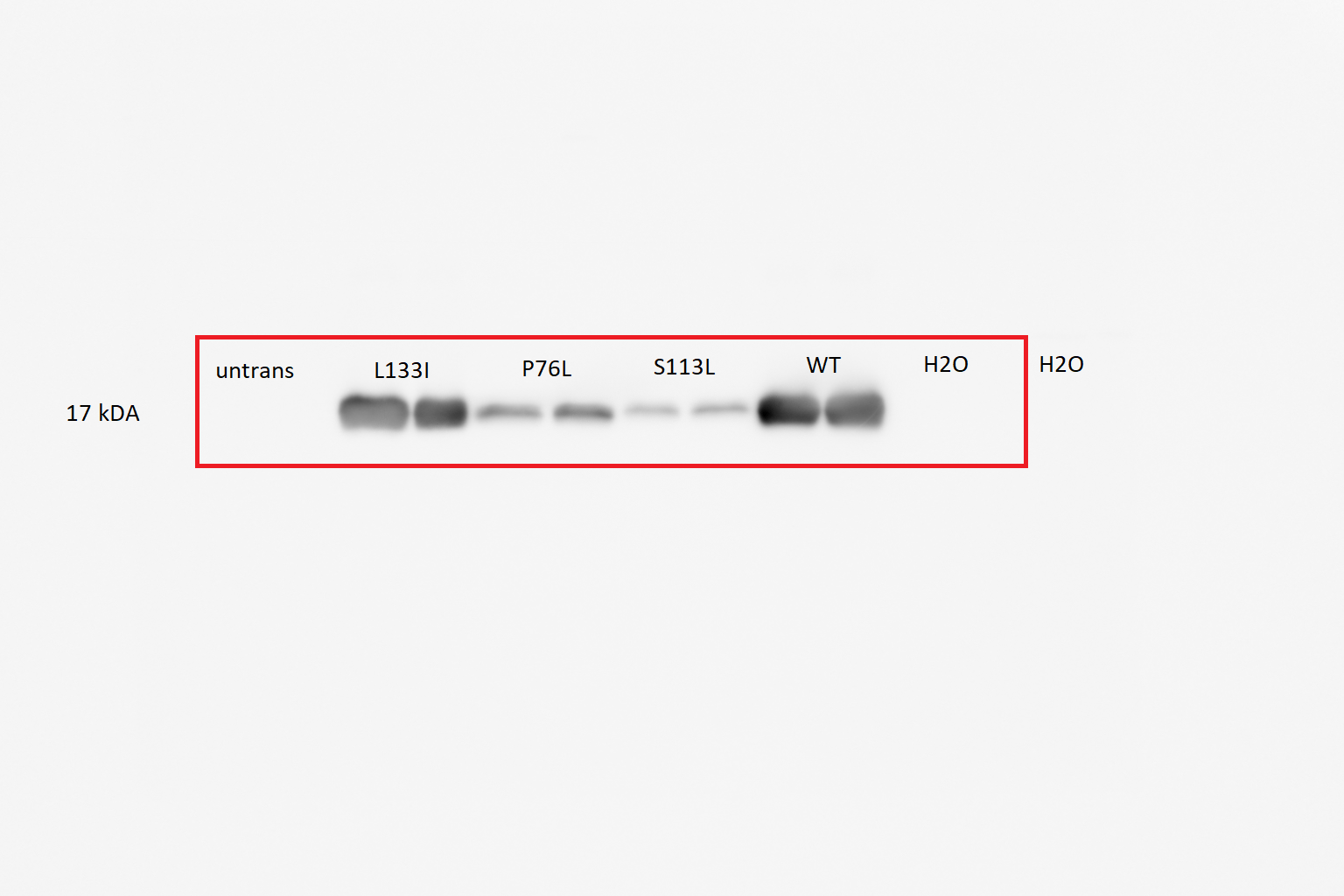

Supplement: Supplementary file 5 — Source data Fig. 3 [file 44321_2025_245_MOESM5_ESM.zip › Figure 3/Figure 3B_IL36RA_mem2.3.tif]

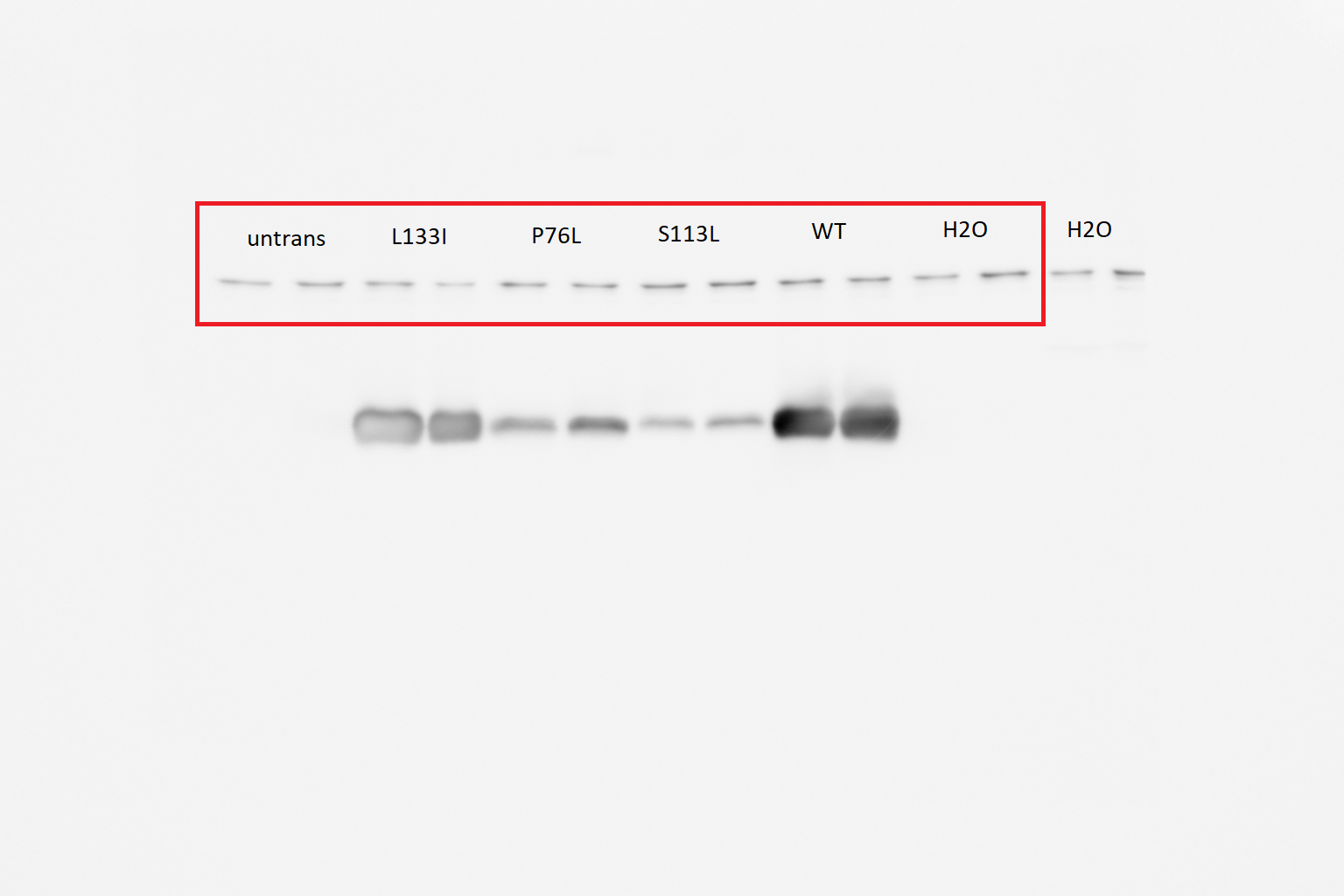

Supplement: Supplementary file 5 — Source data Fig. 3 [file 44321_2025_245_MOESM5_ESM.zip › Figure 3/Figure3B_beta_actin_mem2.3.tif]
