# Supplementary material for: IL-36 signaling as a drug target in Crohn’s disease patients with IL36RN mutations
Source: EMBO Mol Med. 2025 May 30;17(7):1539–55. doi: 10.1038/s44321-025-00245-z (PMC12254353; doi:10.1038/s44321-025-00245-z)
Supplement: Supplementary file 7 — Expanded View Figures [file 44321_2025_245_MOESM7_ESM.pdf]

Expanded View Figures

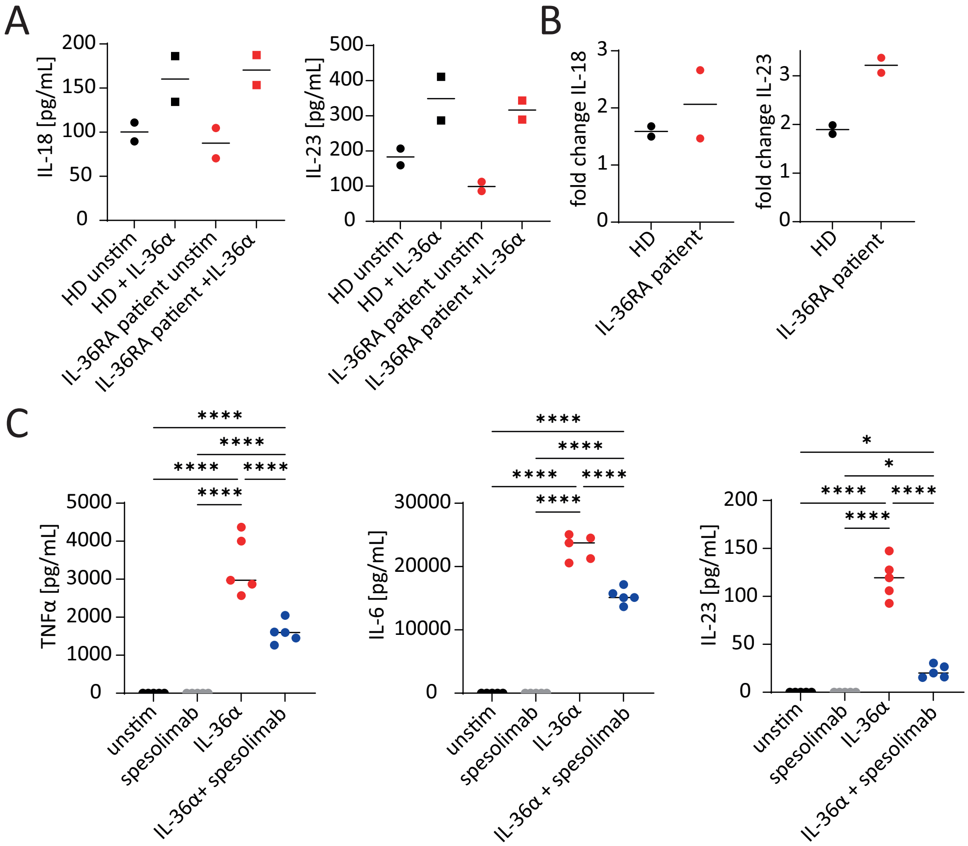

Figure EV1. Induction of pro-inflammatory cytokines by IL-36α stimulation of PBMCs and blockade of cytokine production by the anti-IL-36R antibody spesolimab.

(**A**, **B**) Peripheral blood mononuclear cells (PBMCs) of the *IL36RN*-mutated patient (IL-36RA patient) and one healthy donor (HD) were stimulated in vitro with IL-36α for 7 h or left unstimulated (unstim). Subsequently, cytokine levels in the supernatant were analyzed by cytometric bead array (CBA). Duplicates represent technical replicates. The line in the plots indicates the median. (**B**) Fold change between PBMCs stimulated with IL-36α and those left unstimulated. (**C**) PBMCs of the IL-36RA patient were pre-incubated with 1000 μg/mL spesolimab for 15 min and then stimulated with 100 ng/mL IL-36α for 4 h. Subsequently, the concentration of various cytokines in the supernatant was measured by CBA. Data represent technical replicates. Statistical significance was determined by one-way ANOVA with Tukey's multiple comparisons test. ****$P < 0.0001$, ***$P < 0.001$, **$P < 0.01$, *$P < 0.05$. Exact *P* values for the statistical comparisons are shown in Appendix Table S3. Source data are available online for this figure.

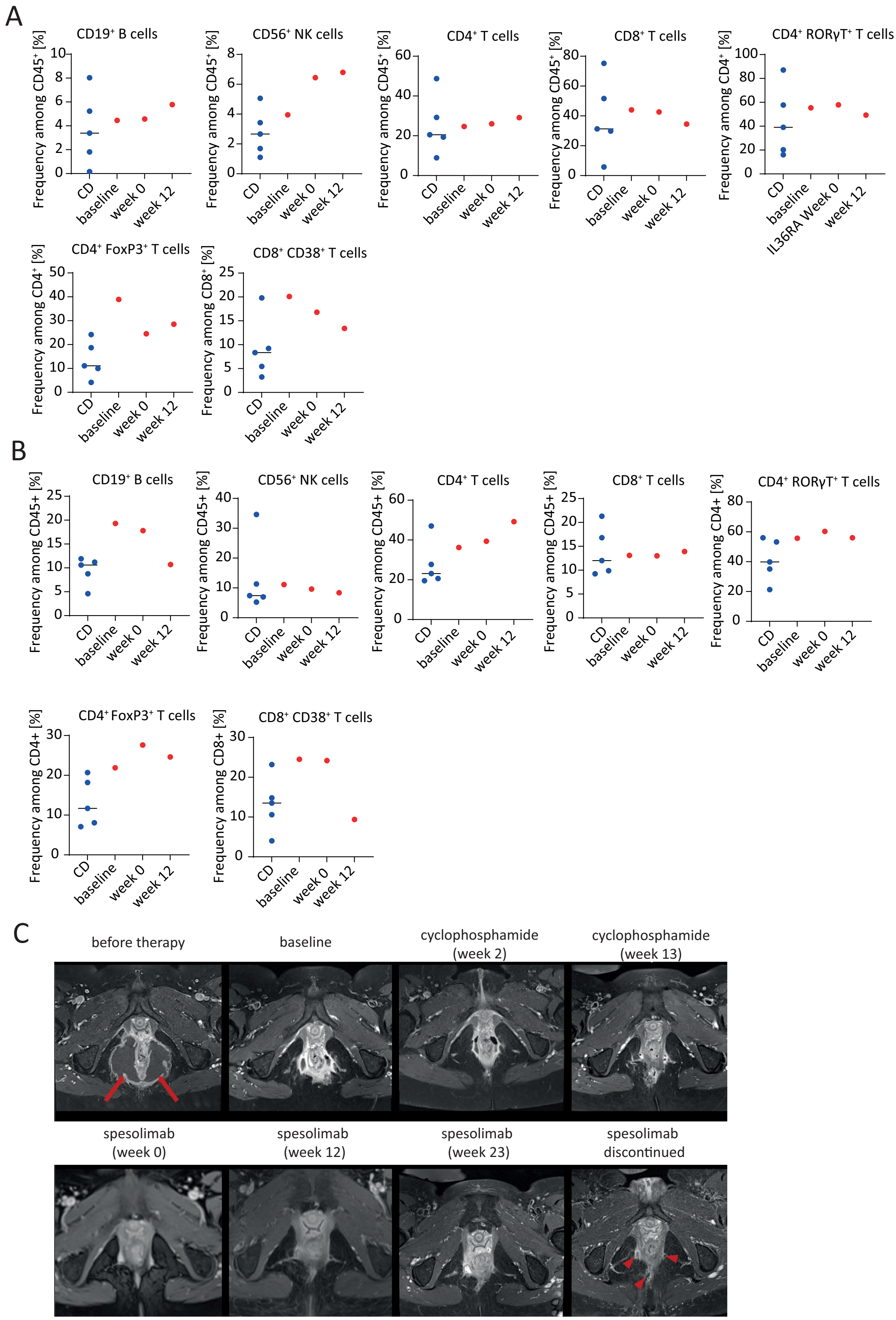
A
CD19⁺ B cells
Frequency among CD45⁺ [%]
CD56⁺ NK cells
Frequency among CD45⁺ [%]
CD4⁺ T cells
Frequency among CD45⁺ [%]
CD8⁺ T cells
Frequency among CD45⁺ [%]
CD4⁺ RORγT⁺ T cells
Frequency among CD4⁺ [%]
CD4⁺ FoxP3⁺ T cells
Frequency among CD4⁺ [%]
CD8⁺ CD38⁺ T cells
Frequency among CD8⁺ [%]
CD
baseline
week 0
week 12
IL36RA Week 0
B
CD19⁺ B cells
Frequency among CD45+ [%]
CD56⁺ NK cells
Frequency among CD45+ [%]
CD4⁺ T cells
Frequency among CD45+ [%]
CD8⁺ T cells
Frequency among CD45+ [%]
CD4⁺ RORγT⁺ T cells
Frequency among CD4+ [%]
CD4⁺ FoxP3⁺ T cells
Frequency among CD4+ [%]
CD8⁺ CD38⁺ T cells
Frequency among CD8+ [%]
CD
baseline
week 0
week 12
C
before therapy
baseline
cyclophosphamide (week 2)
cyclophosphamide (week 13)
spesolimab (week 0)
spesolimab (week 12)
spesolimab (week 23)
spesolimab discontinued

Figure EV2. Effects of spesolimab treatment on immune cell composition in the lamina propria and on abscess healing in the *IL36RN*-mutated patient.

(**A**) Frequency of different immune cell populations in lamina propria mononuclear cells (LPMCs) isolated from ileal biopsies of the *IL36RN*-mutated patient (IL-36RA patient) before and during spesolimab therapy as well as of control Crohn's disease (CD) patients. CD: $n = 5$, IL-36RA patient: $n = 1$. (**B**) Frequency of different immune cell populations in lamina propria mononuclear cells (LPMCs) isolated from colonic biopsies of the IL-36RA patient before and during spesolimab therapy as well as of CD patients. CD: $n = 5$, IL-36RA patient: $n = 1$. (**C**) T1 weighted, fat-saturated MRI after i.v. contrast administration. The ischiorectal fossa is shown in an axial plane. The patient had a horseshoe perianal abscess (arrows) in January 2022, which was surgically relieved and subsequently treated with seton stitches. In the further course up to and including January 2023, the abscess healed under the sequential treatment with cyclophosphamide and spesolimab/certolizumab pegol through scarring (arrowheads). Source data are available online for this figure.

© The Author(s)
