# Supplementary material for: IL-36 signaling as a drug target in Crohn’s disease patients with IL36RN mutations
Source: EMBO Mol Med. 2025 May 30;17(7):1539–55. doi: 10.1038/s44321-025-00245-z (PMC12254353; doi:10.1038/s44321-025-00245-z)
Supplement: Supplementary file 6 — EV Figure Source Data [file 44321_2025_245_MOESM6_ESM.zip › Expanded View Figures/Figure EV2.zip!/Figure EV2/MRI image abscess spesolimab.pdf]

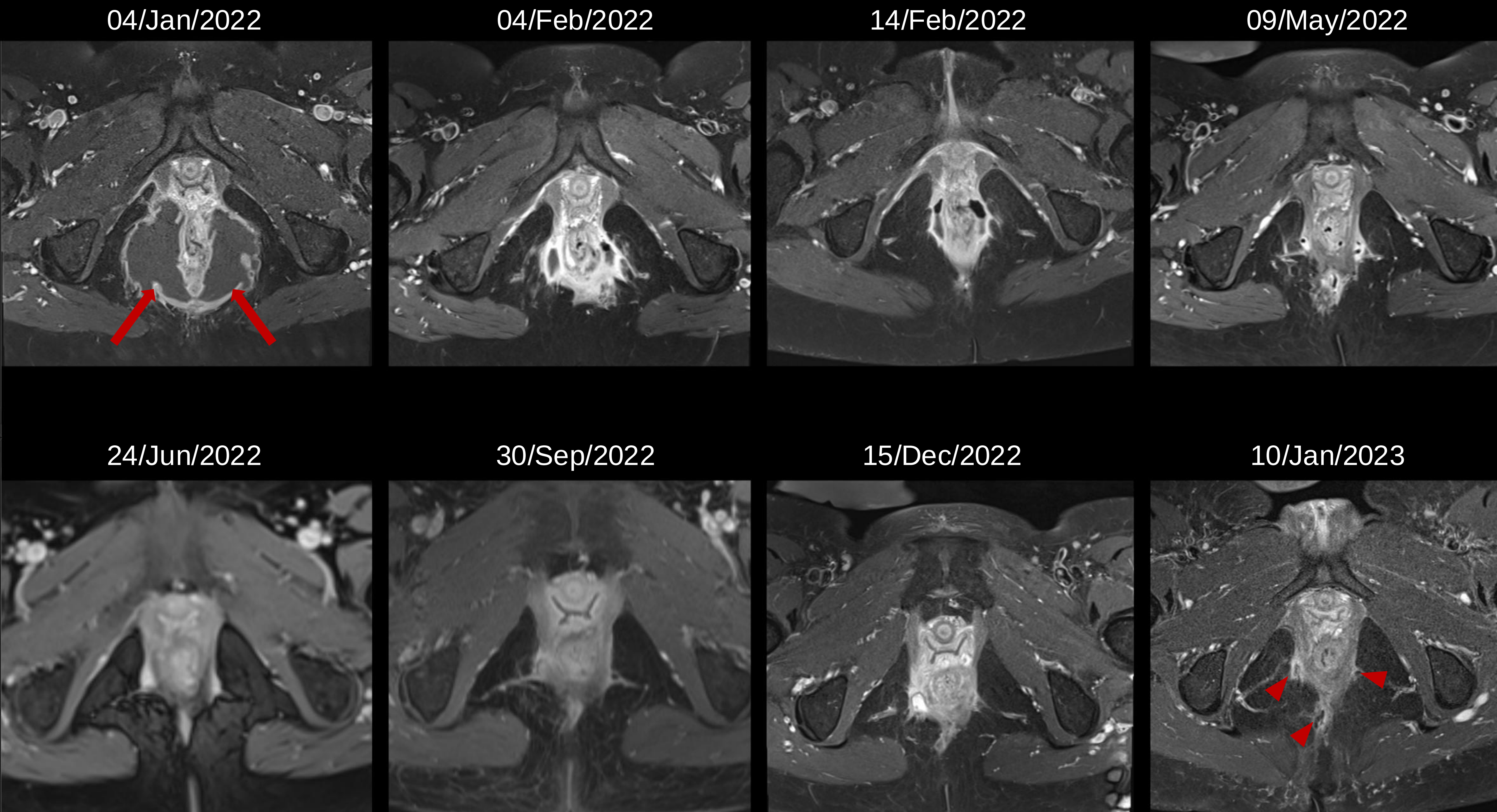
04/Jan/2022
04/Feb/2022
14/Feb/2022
09/May/2022
24/Jun/2022
30/Sep/2022
15/Dec/2022
10/Jan/2023
